# Surveillance Data Highlights Feed Form, Biosecurity, and Disease Control as Significant Factors Associated with Salmonella Infection on Farrow-to-Finish Pig Farms

**DOI:** 10.3389/fmicb.2018.00187

**Published:** 2018-02-15

**Authors:** Hector Argüello, Edgar G. Manzanilla, Helen Lynch, Kavita Walia, Finola C. Leonard, John Egan, Geraldine Duffy, Gillian E. Gardiner, Peadar G. Lawlor

**Affiliations:** ^1^Teagasc, Food Research Centre, Ashtown, Ireland; ^2^Pig Development Department, Animal and Grassland Research and Innovation Centre, Teagasc, Fermoy, Ireland; ^3^School of Veterinary Medicine, University College Dublin, Dublin, Ireland; ^4^Central Veterinary Research Laboratory, Department of Agriculture, Food and the Marine, Backweston, Ireland; ^5^Department of Science, Waterford Institute of Technology, Waterford, Ireland

**Keywords:** control, foodborne-pathogen, risk factors, feed, swine dysentery, biosecurity

## Abstract

Among the zoonotic pathogens affecting pigs, *Salmonella* stands out due to the high number of human cases linked to pork consumption. In the last two decades many countries have put considerable effort into the control of the infection by surveillance and control strategies on farm. Despite this effort, many herds still have a high *Salmonella* prevalence and they require guidance to address this problem. The present study, using the serological surveillance data of finishing pigs from the Irish National pig Salmonella Control Programme, aimed to highlight factors associated with increased risk or that might mitigate *Salmonella* occurrence on farm. A questionnaire with 33 questions regarding herd characteristics, management, feeding, biosecurity, and health was completed for 61 individual herds. After the multivariate analysis by linear regression, nine variables were retained in the final model and linked to herd seroprevalence. Home produced-feed linked to the use of meal showed an eight points reduction in *Salmonella* prevalence compared to purchased feed (*p* = 0.042). Different biosecurity measures were associated to lower seroprevalence. Changing of footwear from outside to inside the farm decreased seroprevalence nearly 20 units (*p* = 0.014) and policies not permitting access to the farmyard to feed trucks (*p* = 0.048) or avoiding the presence of cats on the farm (*p* = 0.05) were estimated in 10 units less of seroprevalence. In contrast, the lack of perimeter fence increased the chance to have higher seroprevalence in five units (*p* = 0.05). Finally, intestinal diseases such as swine dysentery (*p* = 0.044) and *E. coli* diarrhea (*p* = 0.1) were estimated to increase *Salmonella* prevalence in ~20 and 10 units, respectively, demonstrating the importance of controlling other enteric pathogens in an on-farm *Salmonella* control programme. These results show the usefulness of surveillance data to improve on-farm control and confirm that *Salmonella* infection in pigs is multi-factorial and the approach to its control should be multifaceted.

## Introduction

Among food-borne pathogens in the EU, *Salmonella* ranks second in the number of human cases, after *Campylobacter* spp., and is responsible for the highest number of food-borne outbreaks (EFSA, 2015). Pork is one of the main sources of human salmonellosis cases and following the successful implementation of control programmes in poultry, the relative proportion of salmonellosis cases attributed to pork consumption has risen (De Knegt et al., 2015).

Many countries, including Ireland, have surveillance and control programmes in operation which aim to reduce the risk of *Salmonella* transmission in the pig production chain (Quirke et al., 2001; Stärk et al., 2002; Alban et al., 2012). The Irish National Pig *Salmonella* Control Programme (NPSCP) commenced in 2002 and was revised in 2010 with the aim of reducing *Salmonella* prevalence in the pork production chain. Similar to other on farm control programmes (Alban et al., 2012) the NPSCP collects sera (six samples per month) from each herd and the prevalence is estimated considering the results from the last 3 months using a weighting of 3:1:1 with the results from the most recent month having the highest weighting. All herds with a prevalence value over 50% are categorized as high risk and are required to put control measures in place.

On farm control includes potential strategies such as the use of vaccines (Arguello et al., 2013b), organic acids (Arguello et al., 2013a; Walia et al., 2016) and many other potential actions related to husbandry, management, hygiene, biosecurity, and feed (De Busser et al., 2013; De Ridder et al., 2013; Burns et al., 2015). Risk factor studies help to determine which on farm actions may be most effective in reducing on farm prevalence as well as identifying factors likely to increase the risk of having *Salmonella* in the herd. A number of studies to identify such on-farm factors have been performed either using bacteriology (van der Wolf et al., 2001a; Lo Fo Wong et al., 2004; García-Feliz et al., 2009; Correia-Gomes et al., 2012, 2013), or serology (van der Wolf et al., 2001b; Beloeil et al., 2007; Smith et al., 2010). Interestingly factors related to management such as all-in/all-out policy (AI/AO), hygiene, presence of other diseases such as Porcine respiratory and reproductive syndrome (PRRS) and particularly factors associated with feed (coarseness, meal vs. pelleted, home-produced vs. purchased) have been associated with infection, but results are not always consistent across studies, while findings on the effect of factors such as herd size, cleaning protocols, and antimicrobial usage are disputed. These differences may be related to the outcome variable (bacteriology or serology), the type of study (cross-sectional or cohort studies) and even to the serotype or serotypes involved in the infection (Correia-Gomes et al., 2012). Despite the effort, inconsistent results require more research to clarify how to mitigate the on farm *Salmonella* burden. Data from surveillance programmes such as the Irish NPSCP is extremely useful in performing epidemiological studies (Baptista et al., 2010; Smith et al., 2010). The aim of the present study was to provide new insights regarding on farm practices related to herd characteristics management, husbandry, feeding, biosecurity and diseases, that affect the herd *Salmonella* prevalence by the analysis of data from a farm questionnaire combined with serology data provided by the NPSCP.

## Materials and methods

### Questionnaire survey

A cross-sectional study, using a questionnaire survey, was conducted to collect information from Irish herds which sold finisher pigs to the slaughterhouse. Only farrow-to-finish and finishing herds were included in the study as breeding herds (those selling weaner pigs) are not included in the surveillance performed by the NSPCP and the number of such herds is small in Ireland. The questionnaires were completed between October 2014 and May 2015 during workshops with farmers and visits to farms. The questionnaire was designed and tested prior to use in collaboration with Teagasc pig advisors and farm staff. The purpose of the study and instructions on how to complete the questionnaire were explained to the farmers. A cover letter was included with the questionnaire explaining the aim of the study, the confidentiality of the results, the importance of accuracy in filling out the questionnaire, and contact details to obtain clarifications if required. In total, 33 closed questions were posed regarding factors previously included in studies on risk factors for *Salmonella* and adapted to the particularities of pig production in Ireland. The questionnaire was divided into five sections or topics: with questions related to herd characteristics, herd management (Table 1) feed and water (Table 2), hygiene and biosecurity (Tables 1, 3), and herd health (Table 3).

**Table 1:** Description of variables associated to herd characteristics, herd management and biosecurity included in the questionnaire completed by 61 Irish herds.

**Variable**	**Categories**	**No. Herds (%)^a^**
**HERD CHARACTERISTICS**		
Herd size	Number of sows in the herd	Continuous
Other animal species in the herd	No	40 (65.6)
	Cattle	21 (34.4)
	Sheep	1 (1.6)
Full-time staff	No. of people in the herd	Continuous
Permanent staff	Yes	40 (65.6)
	No	21 (34.4)
Labor employed	Yes	42 (68.9)
	No	19 (31.1)
Specialized areas of work	Yes	42 (68.9)
	No	19 (31.1)
Training courses	Yes	32 (52.5)
	No	29 (47.5)
Distribution of production stages (Yes/No)	Weaning	51 (83.6)/10 (16.4)
	Growing	50 (82)/11 (18)
	Finishing divided	13 (21.3)/48 (78.7)
**HERD MANAGEMENT**		
All-in/all-out policy (Yes/No)	Farrowing	48 (78.7)/13 (21.3)
	Weaning	46 (75.4)/15 (24.6)
	Finishing	34 (55.7)/27 (44.3)
Pig regrouping (Yes/No)	Weaning	40 (65.6)/21 (34.4)
	Growing	24 (34.3)/37 (60.7)
	Finishing	22 (36.1)/39 (63.9)
**BIOSECURITY MEASURES**		
Presence of farms within 2 km	Pigs	15 (24.6)
	Cattle	44 (72.1)
	Sheep	14 (22.9)
	Others	1 (1.6)
	No	9 (14.8)
Fence	Single	33 (54.1)
	Double	8 (13.1)
	No	20 (32.8)
Hygienic barrier at the entrance	Yes	11 (18)
	No	50 (81.2)
Loading bay at the entrance	Inside	46 (75.4)
	Outside	14 (22.9)
Access of the feed truck	Inside	13 (21.3)
	Outside	48 (78.7)
Access of the disposal carcass truck	Inside	25 (40.1)
	Outside	36 (59)
Presence of changing room	Yes	47 (81.1)
	No	11 (18.9)
Hygiene and clothes for staff	Hand washing	52 (85.2)/9 (14.8)
	Shower	36 (70.6)/15 (29.4)
	Clothes change	44 (72.1)/17 (27.9)
	Boots change	52 (85.2)/9 (14.8)
Hygiene and clothes for visitors	Hand washing	47 (77)/14 (13)
	Shower	36 (70.6)/15 (29.4)
	Clothes change	46 (75.4)/15 (24.6)
	Boots change	51 (83.6)/10 (16.4)
Policy of visitors (Yes/No)	Require visitors to be free of visiting other *farm*^b^	30 (49.2)/31 (50.8)
Presence of animals on the farm (Yes/No)	Birds	29 (47.5)/32 (52.5)
	Rodents	51 (83.6)/10 (16.4)
	Cats	22 (36.1)/39 (63.9)
	Dogs	18 (29.5)/43 (71.5)

a *Not all questions were answered in all herds, thus not all questions sum 61 farms*.

b *Minimum of 3 days before visiting the herd*.

**Table 2:** Description of the feed variables generated from the questionnaire data completed in 61 Irish herds.

**Variable**	**Categories**	**Farm**	**Sow**	**Weaners**	**Growers**	**Finishers**
Origin	Home-made	–	–	11	14	14
	Purchased	–	–	48	43	47
Type of feed	Liquid	–	4	1	5	4
	Meal	–	30	19	22	30
	Pelleted	–	23	37	30	27
	Dry	–	24	30	18	17
	Wet	–	27	23	32	37
Supplements in feed	Antibiotics	–	–	48/13	8/53	3/58
	Zinc Oxide	–	–	48/13	26/34	1/60
	Acids	–	–	9/52	7/54	8/53
	Whey	–	–	2/59	2/59	7/54
Water supply	Bore hole	46				
	Main supply	12				
	River	1				
	Other	2				
Chlorinated water	Yes	8				
	No	53				
Type of water	Soft	9				
	Hard	42				
	Do not known	10				

**Table 3:** Health and cleaning variables included in the questionnaire data completed in 61 Irish herds^a^.

**Disease**	**No. herds present (%)**	**No. herds free (%)**	**No. herds unknown (%)**
**HEALTH**			
PRRS^b^	29 (47.5)	29 (47.5)	3 (5)
Pleuropneumonia (APP)	20 (32.8)	25 (40.1)	16 (26.2)
Enzootic pneumonia	25 (40.1)	19 (31.1)	17 (27.8)
Glasser	10 (16.4)	31 (50.8)	20 (32.8)
Coccidiosis	17 (27.8)	29 (47.5)	15 (24.6)
PCV2^c^	47 (77)	7 (11.5)	7 (11.5)
Meningitis	32 (52.5)	17 (27.8)	12 (19.7)
Dysentery	6 (9.8)	44 (72.2)	11 (18)
*E. coli* diarrhea	40 (67.8)	10 (16.9)	9 (15.3)
Ileitis	17 (27.8)	22 (36.1)	22 (36.1)
Mange	12 (19.7)	37 (60.6)	12 (19.7)
**Disease complexes**		**No. herds (%) Yes**	**No. herds (%) No**
Respiratory complex (PPRRS|APP|Enzootic pneumonia|Glasser)		50 (81.2)	11 (18.8)
Enteric complex (Dysentery|*E. coli* diarrhea|Ileitis)		49 (80.3)	12 (19.7)
**Protocol**	**Weaning**	**Growing**	**Finishing**
**CLEANING PROTOCOLS**			
No washing	1 (1.7)	8 (17)	14 (23)
Pressurized water	8 (13.1)	2 (4.3)	14 (23)
Detergent	2 (3.4)	2 (4.3)	3 (4.9)
Disinfectant	9 (14.8)	8 (17)	6 (9.8)
Dry	10 (17.2)	6 (12.8)	7 (11.5)
Desiccant	6 (10.3)	5 (10.6)	3 (4.9)
Pressure water + disinfection	15 (25.9)	10 (21.2)	8 (13.1)
Water + disinfection + dry	4 (6.9)	4 (8.6)	4 (6.6)
Water + desiccant	1 (1.7)	2 (4.3)	1 (1.6)
Water + dry + desiccant	3 (5.1)	0	1 (1.6)

a *Not all questions were answered in all herds, thus not all questions sum 61 farms*.

b *Porcine respiratory and reproductive syndrome*.

c *PCV2, Porcine Circovirus2*.

### Salmonella data collection

Meat-juice serological data from Irish herds selling finisher pigs to abattoirs, between January and December 2014, were obtained from the Department of Agriculture Food and the Marine (DAFM), institution responsible for the Irish National pig Salmonella Control Programme. Annual prevalence was estimated by dividing the number of positive pigs delivered to the slaughterhouse in 2014 by the total number of pigs sampled from the same herd during the same period. *Salmonella* serological data were matched to the questionnaire data using the national herd numbers provided in both databases.

### Detection of salmonella (serology)

The detection of antibodies (IgG) in meat juice samples obtained from finishing pigs delivered to the slaughterhouse was performed by an indirect enzyme-linked immunosorbent assay (ELISA). In most cases, six pigs per herd were randomly selected at the slaughterhouse for sampling each month, although frequency of sampling varied occasionally subject to delivery of pigs to the abattoir. Meat juice samples consisted of 10 g of the intercostal muscle. Samples were submitted to the National Reference Laboratory where they were frozen and stored at ~−20°C until analysis. Prior to analysis, each sample was thawed and the muscle fluid was then analyzed by an in-house ELISA based on the Danish mix-ELISA (Nielsen et al., 1998). The indirect ELISA used allows the detection of porcine IgG against the O-chain of the lipopolysaccharide from *Salmonella* serogroups B, C1, and D. Calibrated optical densities (OD%) were obtained by regression analyses of positive and negative reference sera. The meat-juice test was considered positive above a cut-off of 40 OD%. According to Nielsen et al. (1998) the sensitivity of the test is ~89–100% and the specificity is 98–100%, at individual animal level.

### Statistical analysis

A database including information from questionnaires and the NPSCP was created using Excel (Microsoft Office). Any inconsistency in answers was discussed with farmers or Teagasc farm advisors and corrected if necessary. A total of 125 variables were created from the answers obtained in the questionnaire.

All statistical analysis was conducted using SAS 9.3 (Cary, NC). As a first step, a descriptive analysis was performed to identify variables with a large number of missing observations or with low variability making them of little value for further investigation. After this validation step, a univariate analysis was conducted using the annual *Salmonella* herd prevalence as the outcome variable. A relaxed *P*-value ≤ 0.25 was used to select variables for further analysis in a multivariable model. Collinearity was evaluated among pre-selected variables using chi-square and Fischer's tests. From the correlated variables the ones with the lowest *P*-value and/or that made most biological sense were selected for the final multivariable model. Multivariable analysis was performed using a stepwise selection. Variables were retained in the model when *p*-value was <0.15 while α = 0.05 was established as threshold for significance. Interactions were checked among all the variables in the model and introduced one by one to see if they would improve the fitness of the model. As well, all rejected variables were added separately into the final model to ensure no significant variables had been omitted. Two-way interactions were checked among all the variables in the model.

## Results

A total of 67 questionnaires were returned. Six of these were discarded due to the low number of samples tested for *Salmonella* sero-prevalence on these farms throughout 2014. Thus, 61 farms were used for further analysis. In these herds the number of samples analyzed in 2014 varied from 24 to 96, with a median of 72.0 tests analyzed per herd (SE = 1.5). More than 95% of herds had at least 48 tests performed. The mean annual prevalence in 2014 was 25.4 (SE = 2.4). There was no correlation between number of sera analyzed and herd prevalence (*r* = 0.031). Herd prevalence ranged from six herds completely negative in the analysis performed (prevalence 0%) to a maximum prevalence of 79.2% in one herd. A total of 125 variables were derived from the questionnaire circulated to the farmers (Tables 1–3). Of the 61 farms included in the analysis, all but three were farrow-to-finish herds. The three non farrow-to-finish herds were finishing farms which purchased weaner pigs from specialist breeding herds. The mean number of sows per farrow-to-finish herd was 586 and the median was 410 sows/herd (*SD* = 511.7). The smallest herd in the study had 50 sows and only three of the herds included had <100 sows. There was no correlation between herd size and herd prevalence (*R* = 0.03).

A number of variables were removed due to the low variability exhibited in the descriptive analysis of the data. Low variability between herds (defined as ≤3 herds in a category) was detected in variables such as type of herd, replacement policy (few herds purchased weaners and/or finishers), feed allocation (*ad-libitum* access to feed was provided in all the herds) and the use of antimicrobials or pharmacological levels of zinc oxide in finisher feed (not practized in most herds). Thirty-five variables were selected from the univariate analysis with a *P*-value < 0.25 (Table 2).

Collinearity was observed among related variables (Supplementary Table 1). For example, strong collinearity was observed for type of feed (pelleted or meal) in different production stages and feed delivery (dry or liquid feeding) as well as source of feed (home produced or purchased) and feed delivery where all home-produced feed was fed as meal. Similarly, collinearity was observed among variables related to washing protocols. Other variables with collinearity were chlorinated water, turnover of staff in the last 2 years or change of clothes by visitors (Table 4).

**Table 4:** Variables associated with *Salmonella* using a relaxed *p*-value (*P* < 0.25) from univariable mixed linear regression of meat juice ELISA herd prevalence results obtained from slaughtered pigs during 2014.

**Potential factor indicator**	**Level**	**Estimate^1^**	***P*-value**
Staff change^a^^*^,	Yes No	0 −5.968	0.246
Training course^b^^*^^,^^k^	Yes No	0 −8.674	0.069
Origin of weaned pig feed^a^^,^^c^*	Home produced Purchased	−11.963 0	0.05
Origin of growing pig feed ^a,c^^*^	Home produced Purchased	−9.955 0	0.088
Origin of finishing pig feed^a,c^^*^	Home produced Purchased	−0.991 0	0.078
Type feed—sows ^a,c^^*^^,^^g^	Liquid feed Meal Pelleted	−14.465 −22.552 0	0.233
Type feed—weaned pigs^a,c^^*^^,^^g^	Liquid feed Meal Pelleted	−15.215 −11.092 0	0.065
Type feed—finishing pigs^c^^*^,	Liquid feed Meal Pelleted	−14.864 −8.423 0	0.13
Dry or wet feed for weaned pigs^c^^*^	Dry Wet	8.848 0	0.084
Dry or wet feed for growing pigs^c^^*^^,^^d,g^	Dry Wet	8.471 0	0.127
Dry or wet feed for finishing pigs ^c^^*^,	Dry Wet	7.877 0	0.146
Use of whey in finishers	Yes No	0	0.238
Antimicrobials in growing feed^b,c^^*^^,^^e,f^	Yes No	8.892	0.123
Zinc in growing pig feed^c^*^,^^g^,	Yes No	0 −6.189	0.212
Acids in finishing pig feed^d,e^	Yes No	0 −9.833	0.167
Water supply^g^,	Bore hole Main supply River Other	−14.175 −4 to 348 −3.475 0	0.244
Chlorinate water^g^^*^	Yes No	−12.524 0	0.072
Last analysis of water quality	(Months)	–	0.2493
Presence of perimeter fence^f^	No Single Double	4.738 13.637 0	0.0821
Carcass disposal truck^h^^*^	Outside Inside	−10.758 0	0.0252
Feed truck^i^^*^	Outside Inside	−9.195 0	0.1167
Cleaning including disinfection and drying at growing^j^	Yes No	0 −6.711	0.243
Cleaning including pressurized water at finishing^j^	Yes No	0 6.617	0.149
Cleaning including disinfection at finishing^j^	Yes No	0 6.930	0.168
Change of boots by staff^k^	Yes No	0 9.427	0.163
Change of boots by visitors^h,k^^*^	Yes No	0 8.037	0.215
Presence of cats	Yes No	0 −8.628	0.071
Presence of birds^h^	Yes No	0 −5.376	0.243
Glasser's disease^h^	Yes No Unknown	12.628 0 −1.0801	0.051
Coccidia present^i^	Yes No Unknown	6.394 11.906 0	0.128
Swine Dysentery disease	Yes No Unknown	20.811 6.128 0	0.078
*E. coli*^l^ *Diarrhea*	Yes No Unknown	7.522 −4.861 0	0.055
Mange^i^	Yes No Unknown	−1.655 7.6833 0	0.212
Respiratory complex^l^	Yes No	0 −9.330	0.147

1 *Estimate defines the influence of variable levels in the seroprevalence of Salmonella within the herd*.

a–l *Collinearity among selected variables*.

* *Denotes collinearity among the variable with all others with the same letter*.

**Figure 1:**
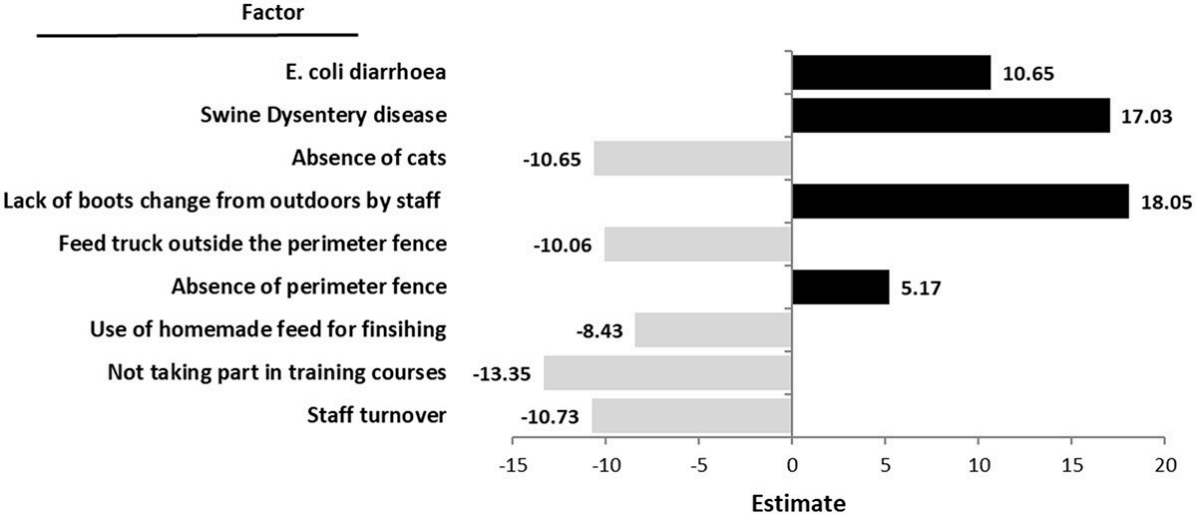
Multivariable mixed linear regression of *Salmonella* sero-prevalence as measured by meat juice ELISA from slaughtered pigs in 61 Irish herds.

Twenty-one variables were included in the multivariate analysis (Table 4). Nine of these variables were retained in the model (Figure 1). Within the feed variables analyzed, farms using home-produced feed were associated to lower seroprevalence compared to those using purchased feed (estimate = −8.42; SE = 4.9; *p* = 0.042). Among biosecurity factors, banning the feed truck access to the farmyard (estimate = −10.06; SE = 4.42; *p* = 0.048), or the absence of cats on the farm (estimate = 10.3; SE = 5.57; *p* = 0.02), exhibited a protective effect to *Salmonella* seroprevalence, while the lack of internal policy to change boots (estimate = 18.05; SE = 6.00; *p* = 0.014), and the lack of perimeter fence (estimate = 13.99; SE = 5.57; *p* = 0.051) were significantly associated to *Salmonella* seroprevalence. Among management factors, those farms without staff turnover within the last 2 years had lower seroprevalence values (estimate = −10.73; SE = 4.28; *p* = 0.042), while those farms without introducing people into training were significantly associated to lower seroprevalence (estimate = −13.34; SE = 4.09; *p* = 0.045). Finally, two diseases were significantly linked to *Salmonella* seroprevalence. Farms with swine dysentery (*Brachyspira hyodysenteriae*) were shown to be increased in their *Salmonella* levels (estimate = 17.02; SE = 7.13; *p* = 0.044) and we also observed a trend for those farms with *E. coli* diarrhea problems (estimate = 10.65; SE = 5.72; *p* = 0.1). None of the interactions among these nine variables was significant.

## Discussion

Among zoonotic pathogens affecting swine, *Salmonella* is the first pathogen associated to human gastroenteritis linked to pork consumption (EFSA, 2015). Pork is ranked as the third most common source of human salmonellosis, but it is at present considered the main source of *Salmonella* from meat in countries where *Salmonella* control in poultry and laying hens has been successful (De Knegt et al., 2015). *Salmonella* control programmes in pig production aim to reduce the burden of *Salmonella* in pork meat. Most of the control programmes include surveillance of the herd status by monitoring the presence of antibodies against *Salmonella* in finishing pigs at market weight (Quirke et al., 2001; Alban et al., 2012). The information garnered from surveillance programmes can be used to categorize herds by risk, but also offers the opportunity to explore the epidemiology and control of the infection (Baptista et al., 2009, 2011; Smith et al., 2010). The present study combining available serological data from the NPSCP database (used as continuous outcome variable) with information gathered through a questionnaire, provided an opportunity to evaluate *Salmonella* risk factors in Irish pig herds. Indirect detection of *Salmonella*, based on the detection of antibodies in the host, offers a number of advantages compared to analysis performed by bacteriology. The fact that antibodies can be detected for long periods of time (Funk et al., 2001) overcomes the problem of the intermittent shedding of *Salmonella* in feces (Beloeil et al., 2003). The surveillance data also offers a huge advantage compared to cross-sectional studies in that the analysis of samples throughout a period of 12 months, allows the level of infection in the herd to be estimated with much more accuracy compared to single values in cross-sectional studies where temporary or seasonal changes may skew the data (Hautekiet et al., 2008). A potential limitation of using surveillance data is the limited number of sera tested per month compared to the number of slaughtered pigs, fact that biases the actual herd prevalence (Nielsen et al., 1998) but with enough power to estimate annual herd prevalence (Alban et al., 2012).

There is vast information in the literature regarding on-farm *Salmonella* risk factors (van der Wolf et al., 2001a,b; Beloeil et al., 2003, 2007; Lo Fo Wong et al., 2004; García-Feliz et al., 2009; Correia-Gomes et al., 2012, 2013). These studies are useful in identifying important factors related to herd characteristics, management, husbandry, hygiene/health that may help prevent or mitigate infection. Conflicting results between studies can be related to particularities of the production system in different countries or by limitations in studies where all factors associated with the infection were not explored. Sixty-one herds were included in the analysis for the present study, which is ~20% of the commercial pig herds in the Republic of Ireland. The number, a good representation of the Irish herds yielded nevertheless a scarce number of surveys compared to previous studies (Kranker et al., 2001; Nollet et al., 2004; García-Feliz et al., 2009) limiting the power of the analysis. To maximize the information gained by the questionnaire, 125 variables were identified from the 33 questions in the survey and of these, nine were retained in the final regression model.

Although feed can be source of *Salmonella* infection (Burns et al., 2015), different studies, including numerous risk factor studies, have also shown that feed form and method of delivery can be used to mitigate *Salmonella* on farm (Mikkelsen et al., 2004; García-Feliz et al., 2009). In agreement with Kranker et al. (2001), the present study found that herds with their own feed mill (home-produced feed) had a lower *Salmonella* prevalence compared to herds purchasing feed. This result is not consequence of the origin of feed but related to the feed presentation (meal or pelleted). Home-made/purchase was included in the model as there were less interactions with other variables compared to meal/pelleted feed variable. All farms with home produced feed, fed meal diets while those purchasing feed were more likely to use pelleted feed. Non-pelleted feed is linked with slower gastric transit time together with a more viscous, porridge-like consistency in the stomach, both of which favor increased microbial fermentation in the stomach (Mikkelsen et al., 2004). Moreover, coarsely-ground meal may not be as well digested as finely ground pelleted feed at the terminal ileum leaving additional carbohydrate substrate to be fermented in the large intestine (Mikkelsen et al., 2004). As a consequence, the growth of lactic acid-producing microbiota is promoted and the concentration of volatile fatty acids is increased, creating a hostile environment for *Salmonella* (low pH, organic acids, competitive exclusion etc.) in the lower gastrointestinal tract (Arguello et al., 2013a).

Factors related to herd characteristics such as herd size, management, and husbandry have been linked with *Salmonella* infection by different studies (Kranker et al., 2001; Vico et al., 2001; Beloeil et al., 2003, 2004; Leontides et al., 2003). All-in/all-out (AIAO) flow disrupts the transmission of infection between production stages (Beloeil et al., 2004; Farzan et al., 2010). However, similar to some other studies (Nollet et al., 2004; Rajić et al., 2007; García-Feliz et al., 2009) our study found no benefit in terms of *Salmonella* mitigation on farms using AI/AO in weaners, growers and finishers. This may be related to the fact that in most instances AI/AO was by room rather than by building as in other studies which is likely to have decreased the effectiveness of the intervention in the current study. Similarly, no potential benefit was demonstrated where cleaning and disinfection protocols were implemented between batches. Nine variables were generated from the survey (Table 1) to analyse the effect of different protocols used (pressurized water, detergent, disinfectant and desiccation) or their combinations. None of them were significant in the final model. As with AI/AO, a reduction in *Salmonella* level would be expected when cleaning protocols are implemented on the farm. However, despite some studies having shown this to be the case (Funk and Gebreyes, 2004), others could not link implementation of cleaning protocols to a decrease in *Salmonella* (Nollet et al., 2004). A possible explanation is that effective cleaning protocols are not correctly performed on farm (Mannion et al., 2007). Among the management and husbandry factors included in the questionnaire, there was a trend for those herds that did not change staff in the previous 2 years to have lower *Salmonella* levels than those where staff turnover was high. This result may be associated with the standard of husbandry on farms, with the possibility of poorer standards on farms with inexperienced staff where staff turnover is highest. Attendance at courses and workshops was linked to a higher prevalence, although we are skeptical of the validity of this result and believe that the question should be revised for further such surveys.

Biosecurity is an essential component in the control of *Salmonella*; external biosecurity decreases the likelihood of introducing *Salmonella* into the herd while internal biosecurity reduces the spread of the infection between stages and batches of pigs (FAO, 2015). The presence of a perimeter fence around the unit and restricting the access of feed trucks to outside the farm yard perimeter from which the feed bins were accessed were two factors linked with a reduced *Salmonella* prevalence in the present study. Similarly, one aspect of internal biosecurity, the change of footwear from outside the unit to inside, was also linked to reduced *Salmonella* prevalence. In addition, the presence of cats on the unit was linked to higher levels of *Salmonella* which agrees with the results of Nollet et al. (2004). Rodents (Vico et al., 2001) were frequently observed in Irish herds in the present study (83.6% of the farmers admitted to seeing rodents on their farms). Cats may help to control rodent populations (Funk et al., 2001) but are themselves a vector for *Salmonella*.

Swine salmonellosis is usually a subclinical infection (Boyen et al., 2008) but severity of infection may be increased by the presence of other infections in the herd. Previous studies have linked the presence of *Salmonella* to other diseases such as PRRS (Beloeil et al., 2007). In the present study we allocated a complete section of the questionnaire to herd health, as we considered that co-infections could be one of the key factors in the perpetuation of the infection over a prolonged period (defined as high prevalence at the end of the year). Our survey included a list of common swine infections, including respiratory, intestinal, and systemic diseases and farmers were instructed to be as precise as possible when indicating the presence or absence of these diseases. Furthermore, questions regarding the vaccination programme used were included in order to gain insight on the pathogens potentially circulating within the herd and their prevention.

Two intestinal disorders were found to be associated with *Salmonella*: swine dysentery and *E. coli* diarrhea. Swine dysentery, a haemorrhagic diarrhea caused by *Brachyspira hyodysenteriae* affects pigs in the growing and/or finishing stages, causing considerable economic losses (Alvarez-Ordóñez et al., 2013). In contrast, *E. coli* diarrhea usually occurs during the suckling or post-weaning periods depending on the pathotype of *E. coli* involved. The strong association between high *Salmonella* prevalence and swine dysentery and the trend toward an association with *E. coli* diarrhea demonstrates the importance of controlling concomitant enteric infections in any *Salmonella* control programme. For example, Walia et al. (2016) attributed the lack of efficacy of an organic acid-based feed additive in controlling *Salmonella* in finishers to the presence of a concomitant *Lawsonia intracellularis* infection (porcine proliferative enteropathy or PPE) and van der Wolf et al. (2001a) linked herds with diarrhea (cause not specified) to presence of *Salmonella*. Intestinal disorders alter the physiological conditions of the gut favoring the development of other pathologies, making it common to find several pathogens during laboratory diagnosis of diarrhea cases (Williamson et al., 2015). The four intestinal pathogens (*Brachyspira* spp., *Lawnonia intracellularis, E. coli*, and *Salmonella*) constitute the basis of the “intestinal complex.” We failed to demonstrate an association between *Salmonella* prevalence and the variable “intestinal complex” which included any of the three other intestinal diseases mentioned above. A potential reason why *Salmonella* and *L. intracellularis* were not linked in the present study despite other studies having associated both pathologies (Borewicz et al., 2015; Walia et al., 2016), is that PPE often causes subclinical disease and farmers may not have been aware of the presence of the pathogen in their herds. However, the fact that two intestinal infectious disorders could be linked to *Salmonella* once again demonstrates the importance of a multifaceted approach in a successful *Salmonella* control programme.

The present study shows the value of surveillance data in uncovering factors associated with on-farm *Salmonella* infection. Feed form (use of meal vs. pelleted) appears to be a useful strategy to mitigate the burden of on-farm *Salmonella*. Biosecurity factors such as perimeter fencing, changing of footwear between outside and inside of the unit and the absence of cats were associated with lower *Salmonella* sero-prevalence, while intestinal diseases (swine dysentery and *E. coli* diarrhea) were linked to higher *Salmonella* sero-prevalence. These results show that *Salmonella* infection in pigs is multi-factorial and highlight that for its control different strategies must be included simultaneously.

## Authors contributions

HA, EM, FL, JE and GD participated in the design of the study. GD, FL, GG and PL provided the funding to perform the study. HA, KW and HL collected the questionnaire data. KW, HL and JE provided the surveillance data. HA, EM, GG and PL performed the analysis of the data. HA, EM, FL, GD, GG and PL wrote the manuscript. All authors approved the final version of the manuscript.

### Conflict of interest statement

The authors declare that the research was conducted in the absence of any commercial or financial relationships that could be construed as a potential conflict of interest.

## Supplementary material

Supplementary Table 1 Collinearity results among selected variables from the univariate analysis in the *Salmonella* risk factors study conducted in 61 Irish farrow-to-finish herds.
